# Laxative Effect of Spicatoside A by Cholinergic Regulation of Enteric Nerve in Loperamide-Induced Constipation: ICR Mice Model

**DOI:** 10.3390/molecules24050896

**Published:** 2019-03-04

**Authors:** Ji Eun Kim, Ji Won Park, Mi Ju Kang, Hyeon Jun Choi, Su Ji Bae, Yusang Choi, Young Ju Lee, Sungbaek Seo, Jin Tae Hong, Dae Youn Hwang

**Affiliations:** 1 College of Natural Resources & Life Science/Life and Industry Convergence Research Institute, Pusan National University, Miryang 50463, Korea; prettyjiunx@naver.com (J.E.K.); pjw08260824@naver.com (J.W.P.); beautifulbead@naver.com (M.J.K.); rudwns546@naver.com (H.J.C.); suji130501@naver.com (S.J.B.); choiyusang@gmail.com (Y.C.); youngju0831@naver.com (Y.J.L.); sbseo81@pusan.ac.kr (S.S.); 2 College of Pharmacy, Chungbuk National University, Chungju 28160, Korea; jinthong@chungbuk.ac.kr

**Keywords:** spicatoside A, constipation, aquaporin, mucin, muscarinic acetylcholine receptors, gastrin, C-kit

## Abstract

Researches on spicatoside A (SpiA)-containing natural products suggest the possibility of SpiA as a potential laxative to alleviate chronic constipation. However, no studies have been conducted with single compound administration of SpiA. To verify the laxative effects and mechanism of action of SpiA on chronic constipation, we investigated alterations in the excretion parameters, histological structure, and cholinergic regulation of the enteric nerve in the colons of Institute of Cancer Research (ICR) mice with loperamide (Lop)-induced constipation after exposure to 20 mg/kg of SpiA. Decrease in the number, weight and water contents of stools in the Lop+Vehicle treated group significantly recovered after SpiA treatment, and alterations in the histological structure and transmission electron microscopy (TEM) images were improved in the Lop+SpiA treated group. Similar recovery effects were observed in the ability for mucin secretion and expression of the membrane water channel gene (aquaporin 8, AQP8). Furthermore, significant improvements were observed in the acetylcholinesterase (AChE) activity and acetylcholine receptors’ (AChRs) downstream signaling pathway after treatment of SpiA. The levels of gastrointestinal (GI) hormones including cholecystokinin (CCK) and gastrin were also remarkably enhanced in the Lop+SpiA treated group as compared to the Lop+Vehicle treated group. The expression of receptor tyrosine kinase (C-kit) and protein gene product 9.5 (PGP9.5) in Cajal and neural cells, as well as the phosphorylation of myosin light chain (MLC) in smooth muscle cells, were recovered after SpiA exposure. Taken together, the results of the present study provide the first strong evidence that SpiA improves chronic constipation through muscarinic cholinergic regulation of the enteric nerve in a Lop-induced constipation ICR mice model.

## 1. Introduction

Spicatoside A (SpiA) belongs to the class of steroidal saponins, which are known complex mixtures having highly branched oligosaccharide moieties and are found to be distributed in various plants [1,2]. They are known to exert a wide range of pharmacological effects including anti-inflammatory, anti-asthma, anti-osteoclastogenesis, neurite outgrowth, memory consolidation and anti-cancer [3]. The anti-inflammatory, anti-asthma and neurite outgrowth activities of SpiA have received great attention since these properties are correlated with chronic human diseases [4,5,6]. Prosapogenin III of SpiA significantly decreases the nitric oxide (NO) production and suppresses the nuclear translocation of nuclear factor (NF)-κB in RAW264.7 cells [4]. SpiA is known to enhance the basal production of mucin 5AC (MUC5AC) mucin in the airway surface epithelium and NCI-H292 cells [5,7,8]. Moreover, it has been shown to stimulate the differentiation and survival of neuronal cells through regulation of neurotrophic factors such as nerve growth factor (NGF) and brain-derived neurotrophic factor (BDNF) [6,9,10]. Although the above studies provide some information regarding the possible correlation between improvement of intestinal bowel diseases and steroidal saponin, no studies have directly investigated the laxative effects of SpiA in the constipated animal model.

Several reports on the effects of extracts and compounds containing saponins have provided evidence for the possibility that steroidal saponin contributes to the decrease of constipation symptoms. Traditional medicines are known to administer saponin containing extracts of *Aloe ferox* Mill. and *Morinda morindoes* for the treatment of constipation [11,12]. Moreover, saponin containing extracts from *Fumaria parviflora* Linn. and *Phyllanthus emblica* fruit showed enhanced movement of charcoal meal and the recovery in the total number of feces, depending on the concentration administered to mice [13,14]. Furthermore, the crude extract of *Viola betonicifolia* containing alkaloid and saponins, and the aqueous extracts of *Liriope platyphylla* containing isoflavone and saponin, also effectively decreased the symptoms of constipation in the loperamide (Lop)-induced model [14,15]. Similar laxative effects have recently been observed in Lop-induced constipation rats treated with the red *L. platyphylla* extract (EtRLP) containing SpiA [16]. However, no study has showed direct evidence of the laxative effects and molecular mechanism of SpiA when administered as a single compound.

Therefore, the present study investigated the possibility that, of the various steroidal saponins, SpiA is a key component for inducing laxative effects in the Lop-induced constipation Institute of Cancer Research (ICR) mice model. We believe that our results provide the first scientific evidence that SpiA successfully improves functional regulation of the neural, Cajal, smooth muscle and epithelial cells in the transverse colon of the Lop-induced constipation ICR mice model.

## 2. Results

### 2.1. Effect of SpiA Treatment on the Stool Excretion Parameters

**Figure 1:**
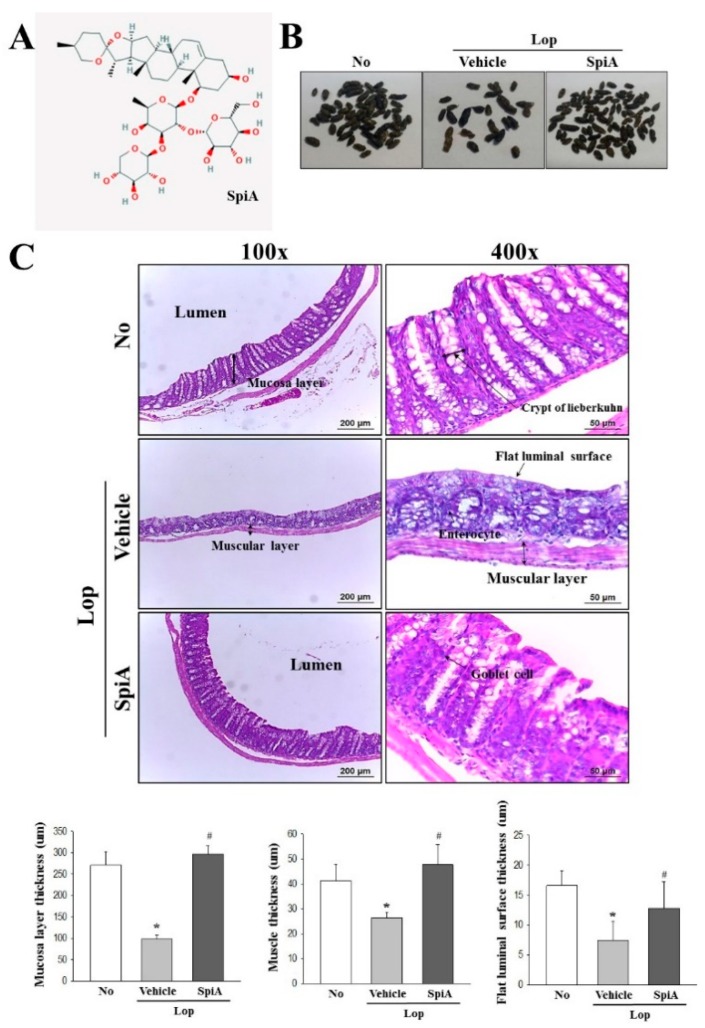
Stools image and histological structures of colon in Lop-induced constipation ICR mice model after SpiA treatment. (**A**) Chemical structure of SpiA. (**B**) Stool images of subset groups. The number of stools were measured in No, Lop + Vehicle and Lop + SpiA treated groups. Five to six mice per group were used in collection of stools. (**C**) H&E stained sections of colon from the No, Lop + Vehicle or Lop + SpiA treated groups were observed at 100× (left column) and 400× (right column) magnification using a light microscope. Five to six mice per group were used in preparation of colon tissue slide and three parameters were assayed in duplicate in two H&E stained slide. Histopathological parameters were determined using the Leica Application Suite (Leica Microsystems). Data are reported as the mean ± SD. *, *p* < 0.05 compared to the untreated group. ^#^, *p* < 0.05 compared to the Lop + Vehicle treated group. No, Untreated group; Lop, Loperamide; SpiA, Spicatoside A, ICR; Institute of Cancer Research, H&E; Hematoxylin and Eosin.

To investigate the beneficial effects of SpiA on stool excretion-related parameters, we investigated alterations in the body weight, food intake, water consumption, stool number and urine volume in Lop-induced constipation ICR mice model after administering SpiA. The number, weight and water content of stools were lower in the Lop + Vehicle treated group than the untreated group. However, the decrease in stool excretion parameters were remarkably recovered in the Lop + SpiA treated group when compared to the Lop + Vehicle treated group (Figure 1B and Table 1). Also, a round form of hard stools of Lop + Vehicle treated group were changed into a longish form of soft stools in the Lop + SPA treated group (Figure 1B). The urine volume in the Lop + SpiA treated group showed a reversal in the pattern from the stool excretion parameters, while no significant differences in body weight and food intake were observed (Table 1). The above results suggest that SpiA treatment stimulates the excretion of stools in the Lop-induced constipation ICR mice model, regardless of the body weight, food intake and water consumption.

### 2.2. Effect of SpiA Treatment on the Histology and Cytology of Colon

**Figure 2:**
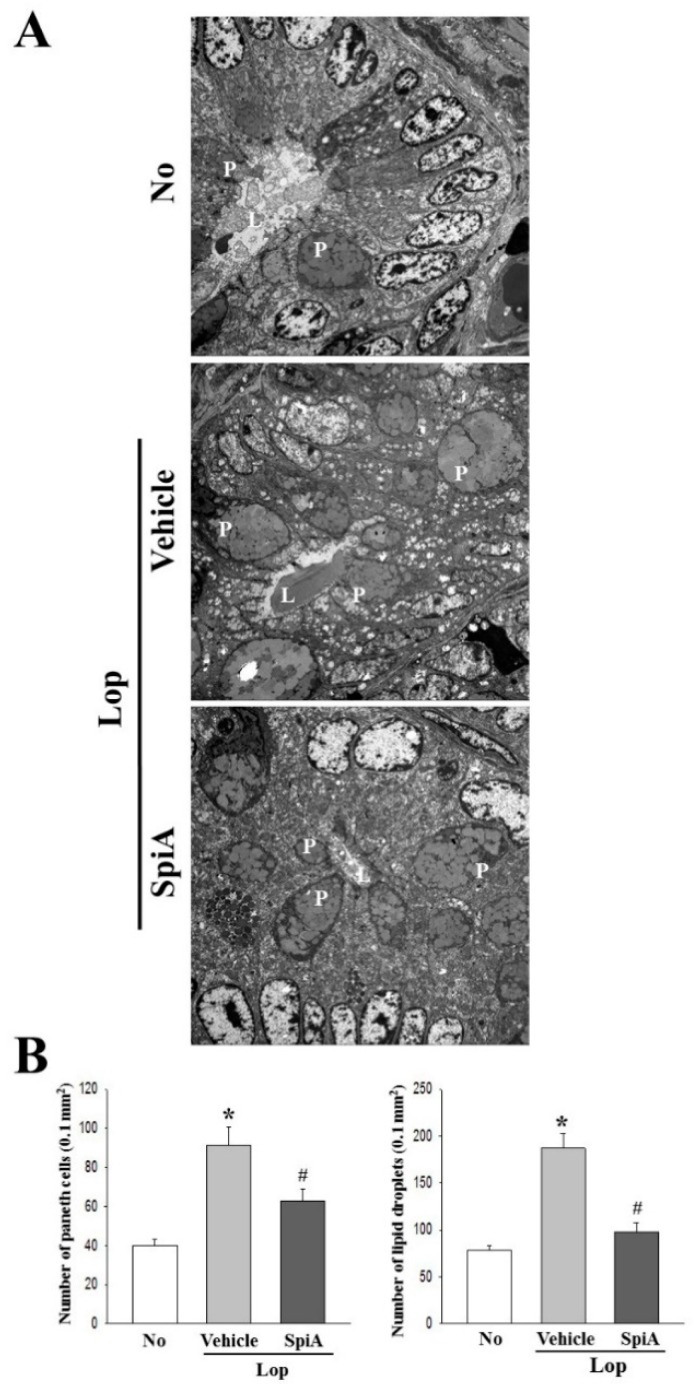
TEM images of colons of mice. (**A**) Crypt ultrastructure of colon in the No, Lop + Vehicle and Lop + SpiA treated groups were observed by TEM at 1800× magnification. (**B**) Crypt lumen diameter, number of Paneth cells, and number of lipid droplets were determined using the Leica Application Suite software. Four to five mice per group were used in preparation of TEM slide and two parameters were assayed in duplicate in each test. Data are reported as the mean ± SD. *, *p* < 0.05 compared to the untreated group. ^#^, *p* < 0.05 compared to the Lop + Vehicle treated group. No, Untreated group; Lop, Loperamide; SpiA, Spicatoside A; TEM, transmission electron microscopy; L, crypt lumen; P, Paneth cells.

To examine whether enhancement of stool excretion induced by SpiA treatment reflects on structural alterations in the colon of the Lop-induced constipation ICR mice model, changes in the histological and cytological structure were observed in colons sections stained with H&E solution and analyzed with transmission electron microscopy (TEM), respectively. In the histological analysis, a significant decrease in the thickness of mucosa, muscle and flat luminal surface was detected in the Lop + Vehicle treated group relative to the untreated group. However, following treatment with SpiA, the three parameter levels dramatically increased when compared with the Lop + Vehicle treated group (Figure 1C). In TEM analysis, Lieberkuhn crypt of the untreated group showed the ring structure in which a central lumen is surrounded by enterocytes, goblet cells and Paneth cells. These structures of the crypt were significantly altered in the Lop + Vehicle treated group, namely, an increase in the number of Paneth cells, lipid droplets and the average size of goblet cells. However, these changes were significantly recovered after Lop + SpiA treatment, although their rate was varied (Figure 2). Taken together, the above results indicate that the enhanced stool excretion after SpiA administration completely reflects an alteration in the histological and cytological structures of the colons in the Lop-induced constipation ICR mice model.

### 2.3. Effect of SpiA Treatment on Dysfunction of the Ability to Secrete Mucin

**Figure 3:**
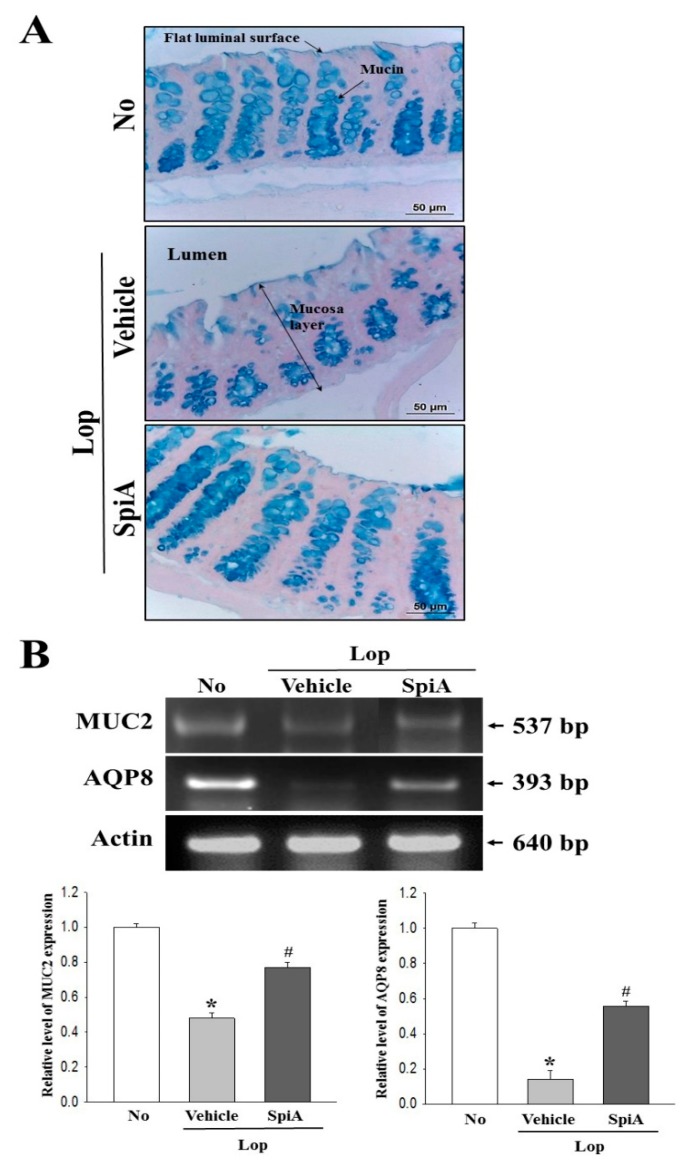

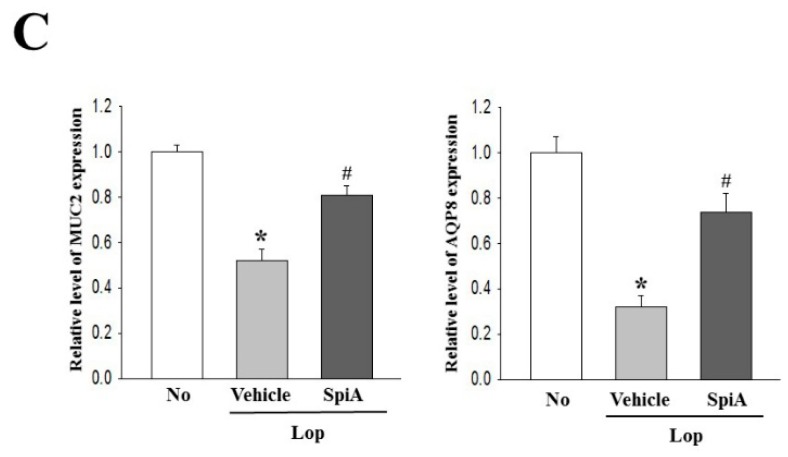
Mucin secretion and membrane water channel expression in the colon. (**A**) Mucin secreted from crypt layer cells was stained with Alcian blue at pH 2.5, and images were observed at 400× magnification. Five-to-six mice per group were used in preparation of Alcian blue stained colon tissue. (**B**) The levels of MUC2 and AQP8 transcripts in the total mRNA of colons were measured by semiquantitative RT-PCR using specific primers. After the intensity of each band was determined using an imaging densitometer, the relative levels of MUC2 and AQP8 mRNA were calculated based on the intensity of actin as an endogenous control. Five to six mice per group were used in preparation of total RNA and specific transcript level was assayed in duplicate in each test. (**C**) Quantitative real-time PCR analyses were performed to evaluate the levels of MUC2 and AQP8 transcripts in the similar way described as described in Figure 3B. Data are reported as the mean ± SD. *, *p* < 0.05 compared to the untreated group. ^#^, *p* < 0.05 compared to the Lop + Vehicle treated group. No, Untreated group; Lop, Loperamide; SpiA, Spicatoside A, RT-PCR; Reverse transcription-polymerase chain reaction, MUC2; mucin 2, AQP8; aquaporin 8.

Next, we investigated whether SpiA treatment recovers the dysfunctional ability to secrete mucin. To achieve this, the levels of mucin secretion and regulatory protein expression were examined in the colon of Lop + SpiA treated mice. The mucosal layer of the colon was stained as dark blue in the crypts of the untreated group. Under conditions of constipation, their levels were decreased as compared to the untreated group. However, the levels of mucin were remarkably increased in the Lop + SpiA treated groups, while the thickness of crypt was enhanced in the same group (Figure 3A). Furthermore, the expression levels of mucin 2 (MUC2) mRNA correlated with the mucin staining pattern, although a few differences were observed in the recovery rate of each group (Figure 3B). We also examined the expression of the membrane water channel to investigate the effect of SpiA treatment on the transmembrane osmotic gradient. Decreased levels of aquaporin 8 (AQP8) mRNA in the Lop + Vehicle treated group were significantly enhanced (375%) in thr Lop + SpiA treated group (Figure 3B). Also, a similar recovery pattern was observed in quantitative real-time PCR analyses (Figure 3C). These results indicate that the excretion stimulating effect of SpiA may be linked with improvement in the mucin secreting ability and membrane water channel expression in the colon of Lop-induced constipated ICR mice model.

### 2.4. Effect of SpiA Treatment on the Cholinergic Regulation of Enteric Neurons

**Figure 4:**
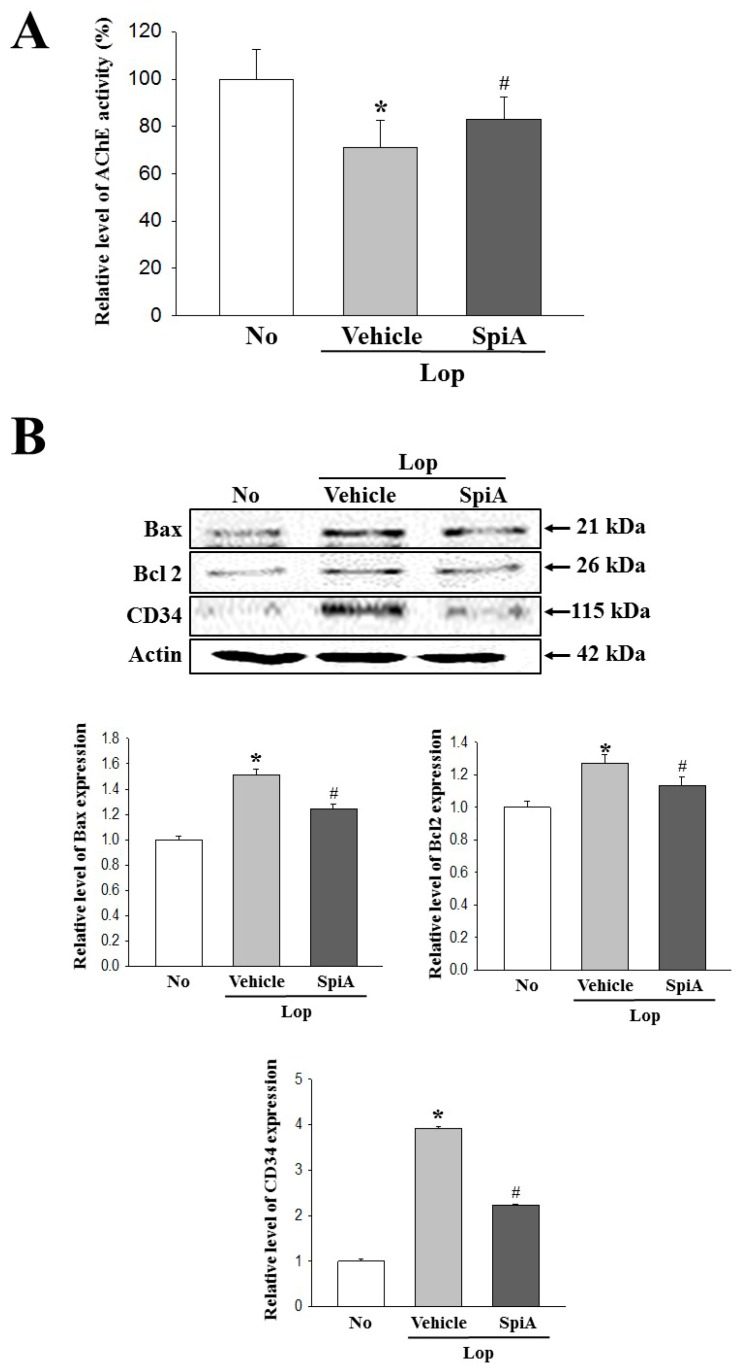
Measurement of AChE activity. (**A**) After homogenization of colon tissue, AChE activity was measured using an Acetylcholinesterase Assay Kit that could detect as little as 0.01 mU AChE in a 100 μL assay volume (0.1 mU/mL). Five to six mice per group were used in preparation of tissue lysate and enzyme activity was assayed in duplicate in each test. (**B**) The expression of Bax, Bcl-2 and CD34 protein representing apoptotic phenomena were measured by Western blot analysis using horseradish peroxidase (HRP)-labeled anti-rabbit IgG antibody. Five-to-six mice per group were used in preparation of total lysate and specific protein level was assayed in duplicate in each test. Data are reported as the mean ± SD. *, *p* < 0.05 compared to the untreated group. ^#^, *p* < 0.05 compared to the Lop + Vehicle treated group. No, Untreated group; Lop, Loperamide; SpiA, Spicatoside A; AChE, Acetylcholinesterase, HRP; Horseradish peroxidase.

**Figure 5:**
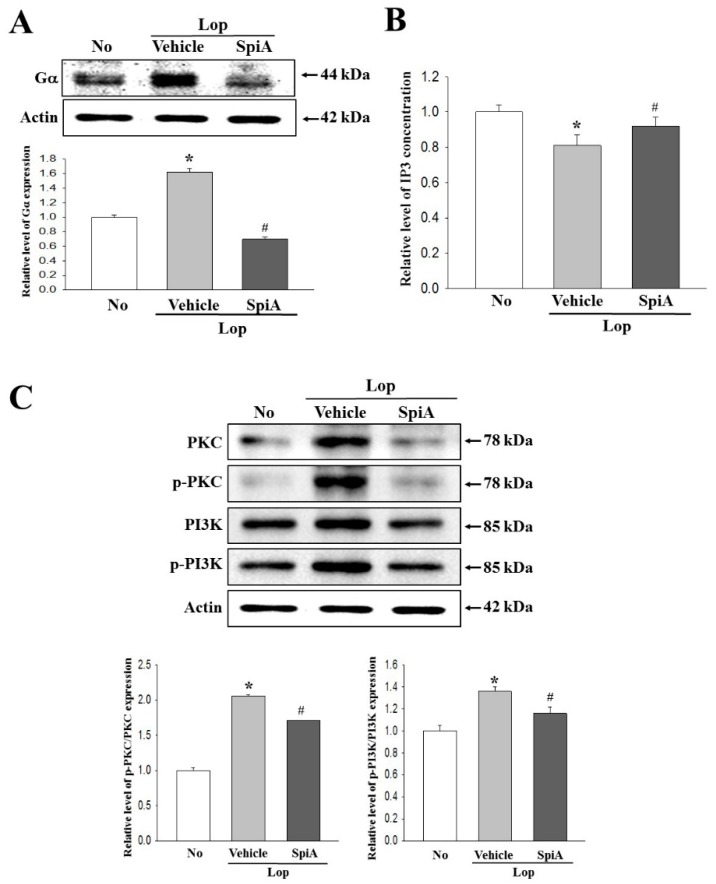
mAChR downstream signaling pathway in the colon treated with SpiA. (**A**) The expression of Gα in the mAChR-signaling pathway were measured by Western blot analysis using HRP-labeled anti-rabbit IgG antibody. Five to six mice per group were used in preparation of total lysate and specific protein level was assayed in duplicate in each test. (**B**) After collection of the colon from mice of each group, tissue lysates were prepared as described in the materials and methods. The IP3 concentration in the total cell lysate was measured using an ELISA kit that detects IP3 at 5 pg/mL to 1000 pg/mL. Five to six mice per group were used in preparation of total lysate and IP3 level was assayed in duplicate in each test. (**C**) The expression of several related proteins, including PKC, p-PKC, PI3K and p-PI3K in the mAChR M2 and M3 signaling pathway were measured by Western blot analysis using HRP-labeled anti-rabbit IgG antibody. After determining each band intensity using an imaging densitometer, the relative levels of the four proteins were calculated based on the intensity of actin. Five-to-six mice per group were used in preparation of total lysate and specific protein level was assayed in duplicate in each test. Data are reported as the mean ± SD. *, *p* < 0.05 compared to the untreated group. ^#^, *p* < 0.05 compared to the Lop + Vehicle treated group. No, Untreated group; Lop, Loperamide; SpiA, Spicatoside A; IP3, Inositol triphosphate, mAChR; Muscarinic acetylcholine receptors, HRP; Horseradish peroxidase, PKC; **Protein kinase C**, PI3K, Phosphoinositide 3-kinases.

The major process of gastrointestinal (GI) tract, including motility, secretion, digestion and circulation, are controlled by the enteric nerve system, although harmonization of the central nervous system (CNS), enteric nervous system (ENS) and GI peptide are considered to be the most important factors [17]. To investigate the role of acetylcholine (Ach) and acetylcholine receptors (AChRs) for GI motility and the ability to secrete mucin during the laxative action of SpiA, alterations in the acetylcholinesterase (AChE) activity and mAChRs downstream signaling pathway were examined in the colon of the subset groups. The decreased AChE activity of Lop + Vehicle treated group was enhanced by 15.3% in the Lop + SpiA treated group (Figure 4A). Also, a reverse regulation pattern was observed in the Ach-regulated apoptotic phenomena. The expression of three apoptotic proteins (Bax, Bcl2 and CD34) were decreased in the Lop + SpiA treated group as compared to the Lop + Vehicle treated group (Figure 4B). Among the members of mAChR downstream signaling pathway, the expression level of Gα and the phosphorylation of protein kinase C (PKC) and phosphoinositide 3-kinase (PI3K) were significantly decreased after SpiA administration (Figure 5A,C). However, IP3 concentration was differed from the above observed patterns of the three mediators. The treatment of SpiA induced the increase of IP3 concentration in the Lop-induced constipation ICR mice model (Figure 5B). Therefore, these results indicate that SpiA plays a key role in ACh secretion, Ach-regulated apoptotic phenomena and the downstream signaling pathway of mAChRs in the colon of Lop-induced constipation ICR mice model when exerting its laxative effects.

### 2.5. Effect of SpiA Treatment on the Regulation of GI Hormone

**Figure 6:**
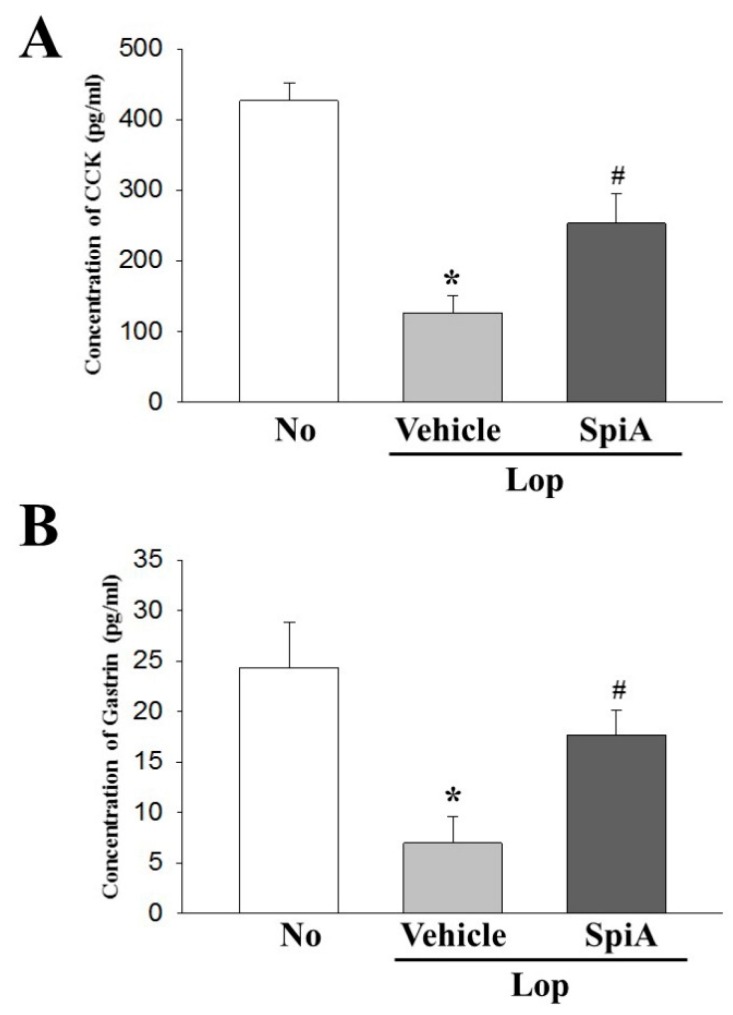
Alterations in the concentrations of CCK and gastrin in the colon of Lop + SpiA treated group. (**A**) The CCK concentration was measured in the colon homogenate by an enzyme-linked immunosorbent assay. The minimum detectable concentration of this kit is 0.1–1000 pg/mL CCK. (**B**) The gastrin concentration was measured in the colon homogenate by an enzyme-linked immunosorbent assay. The minimum detectable concentration of this kit is 0.312–20 pg/mL. Five-to-six mice per group were used in preparation of total lysate and hormone level was assayed in duplicate in each test. Data are reported as the mean ± SD. *, *p* < 0.05 compared to the untreated group. ^#^, *p* < 0.05 compared to the Lop + Vehicle treated group. No, Untreated group; Lop, Loperamide; SpiA, Spicatoside A, CCK; Cholecystokinin.

To examine whether the cholinergic regulation of SpiA is accompanied with alterations of the GI hormones, the concentrations of GI hormones were measured in the homogenate of the colon after SpiA exposure. The concentration of CCK and gastrin were lower in the Lop + Vehicle treated group than the untreated group, which were observed to significantly increase after SpiA treatment in the Lop-induced constipation ICR mice model, although their levels did not reach to those of the untreated group (Figure 6). These data indicate that the cholinergic regulation of SpiA may be associated with the recovery of the GI hormone concentrations in the colon tissue.

### 2.6. Effect of SpiA Treatment on the Function of Neural, Cajal and Smooth Muscle Cells

**Figure 7:**
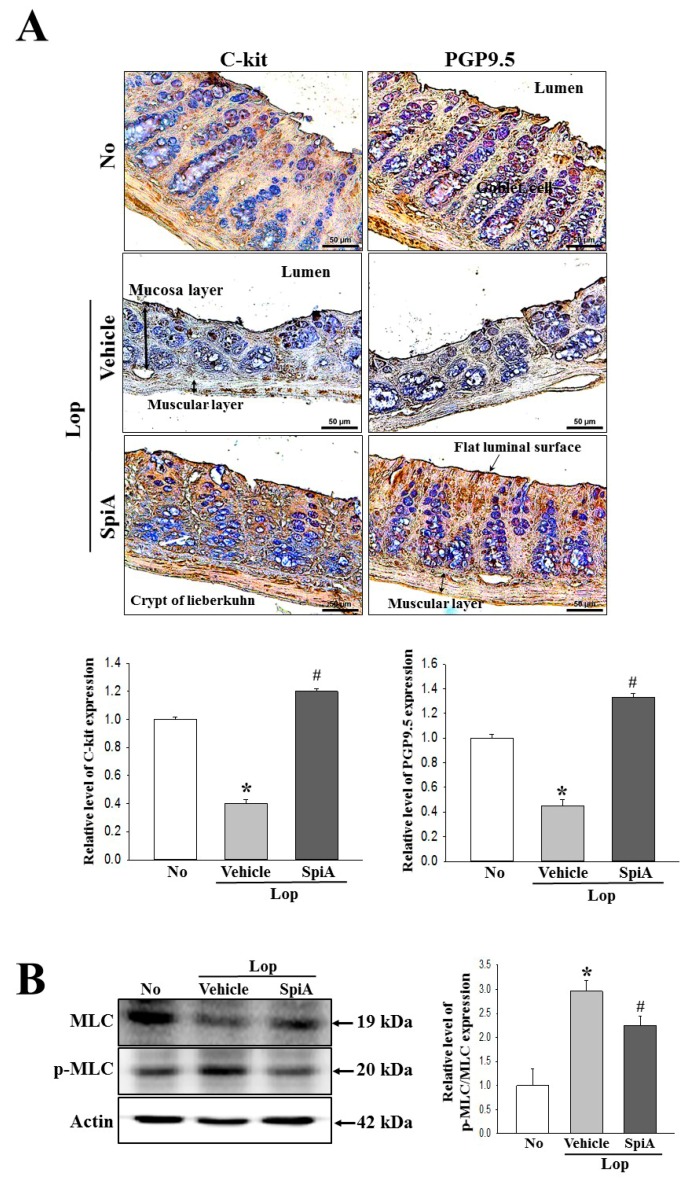
Expression of c-Kit and PGP9.5 and phosphorylation of MLC in neural, Cajal and smooth muscle cells. (**A**) Following staining with c-Kit and PGP9.5 antibody, their levels around the mucosa were observed in colon tissue at 400× magnification. Five to six mice per group were used in preparation of colon tissue slide and expression level was assayed in duplicate in each test. (**B**) Expression of MLC and p-MLC measured using western blot analyses with an HRP-labeled secondary anti-rabbit IgG antibody, and the relative levels of each protein was calculated relative to the intensity of actin bands. Five-to-six mice per group were used in preparation of total lysate and specific protein level was assayed in duplicate in each test. Data are reported as the mean ± SD. *, *p* < 0.05 compared to the not-treated group. ^#^, *p* < 0.05 compared to the Lop + Vehicle treated group. No, Untreated group; Lop, Loperamide; SpiA, Spicatoside A, MLC; Myosin light chains.

Finally, we investigated whether the cholinergic regulation of SpiA accompanies the regulation of the function of neural, Cajal and smooth muscle cells during the laxative effects. To achieve these, the immunoreactivity of C-kit (a marker for cajal cells) and PGP9.5 (a marker for neural cells), and the phosphorylation of Myosin light chains (MLC) (a marker for neural cells) were measured in the colon of Lop + SpiA treated ICR mice. The expression levels of C-kit and PGP9.5 in the Lop + Vehicle treated group were lower than that observed in the untreated group. However, after SpiA exposure, these levels were enhanced in the colon of Lop-induced constipation mice (Figure 7A). Also, a reverse pattern was detected in the phosphorylation of MLC, an indicator for the functioning of smooth muscle cells, which showed significantly decreased levels in the Lop + SpiA treated group (Fiugre 7B). These results suggest that the cholinergic regulation of SpiA may be associated with improving the regulation on the functioning of Cajal and smooth muscle cells during laxative effects in Lop-induced constipation ICR mice model.

## 3. Discussion

Laxative effects to decrease constipation symptoms were investigated in phytochemicals-containing products of several herbal plants without any significant toxicity [13,14]. Among these, saponin-containing products such as *L. platyphylla*, Red *L. platyphylla*, *Aloe ferox, F. parviflora* and *P. emblica* can be considered as potential candidate because they significantly recovered the stool excretion, intestinal motility, mucin secretion and histological structure in Lop-induced constipation model [11,13,14,15,18]. In this study, we investigated the laxative effects and mechanism of action of SpiA, based on previous studies reporting that the SpiA containing red *L. platyphylla* alleviated symptoms in the Lop-induced constipation ICR mice model [19]. We believe that our results of the current study are the first to provide direct evidence that SpiA stimulates stool excretion, recovery of histological and cytological structures, and mucin secretion in Lop-induced constipation ICR mice model. These results especially indicate that the laxative effects of SpiA are tightly correlated with the cholinergic regulation of enteric nerve in the colon of Lop-induced constipation ICR mice model.

Determining the therapeutic dose of drug candidates is considered an important parameter, since these data can be provided as basic information to predict the clinical therapeutic index (TI) of a drug candidate at an early stage [20]. Various concentrations of saponin-containing laxatives have actually been used to evaluate their efficacy in animal model. Among these, 100–300 mg/kg is determined as the optimal concentration of saponin containing extracts of *Aloe ferox, F. parviflora* and *P. emblica* for laxative effects [13,14,18]. Other natural products such as *L. platyphylla* and red *L. platyphylla* extracts which prepared with nine repetitions of a two-step process (steaming 200 g of dry root samples at 99 °C for 3 h after air-drying at 70 °C for 24 h) show an optimal laxative effect at 1000 mg/kg [15,19]. Meanwhile, quercetin as a single compound has been administered at relative low concentrations (10, 20 and 40 mg/kg) [17]. In this study, 20 mg/kg of SpiA was used as the optimal concentration, based on the concentration of quercetin used in a previous study. This dose was the equivalent to 1.62 mg/kg daily dose in humans, according to the Food and Drug Administration in USA (Guidance for Industry Estimating the Maximum Safe Starting Dose in Initial Clinical Trials for Therapeutics in Adult Healthy Volunteers).

The laxative effects of constipation model could be induced by natural products containing various phytochemicals such as saponins, flavonoids, tannins, sterols, terpenoids, alkaloids, phenolic compounds [13,14]. Of these, some flavonoids such as naringenin and quercetin induce the enhancement of stool excretion and mucin secretion in the Lop-induced constipation model [17,21]. Saponins have been investigated to relate with the regulation of smooth muscle contraction in the intestine. Rhizoma Parisdis total saponins (RPS) are known to remarkably inhibit the gastric antral smooth muscle contractility through regulation of muscarinic receptors, while SpiA-containing EtRLP improves the symptoms of Lop-induced constipation in SD rats via the regulation of the mAChR downstream signaling pathway and the ER stress response [22]. The current study is the first to administer SpiA as a single compound into Lop-induced ICR constipation model to verify the laxative effect and mechanism of action. Various effects representing the improvement of constipation were observed after treatment of SpiA, with most outcomes being similar to previous studies. Therefore, we believe that our results are the first direct evidences regarding the molecular mechanism of SpiA exerting its laxative effects in Lop-induced constipation ICR mice model.

The digestive system is controlled by complex mechanisms regulating motility and secretion. During these processes, CNS, ENS and GI peptides participate as key members to control the function of the enteric nerve, Cajal cells, smooth muscle cells and epithelial cells [23]. Some natural products with laxative effects induce the recovery of GI metabolic components and enteric nerve-related factors. *Lactobacillus fermentum* Suo (LF-Suo) significantly increase the serum levels of MTL, gastrin (GAS), endothelin (ET), AChE, substance P (SP) and vasoactive intestinal peptide (VIP) as well as the expression levels of c-Kit, SCF and GDNF during the preventive effects for constipation in mice model [24]. Also, a similar effect was observed in the Lop-induced constipation model after administering aqueous extracts of *Herba Cistanche* and naringenin [25,26]. Furthermore, gallotannin-enriched extracts isolated from Galla Rhois (GEGR) successfully improves the concentration of four GI hormones, including CCK, GAS, somatostatin (SS) and MTL, and their receptor signaling pathways in the constipated animal model [27]. In the current study, we measured the levels of GI metabolic components and enteric nerve-related factors in the Lop + SpiA treated group to investigate the molecular mechanism of SpiA on laxative activity. Our results are consistent with above previous findings, although there are some differences in the analyzed factors. These results therefore provide novel evidences that SpiA contributes to improving the level of GI metabolic components and enteric nerve-related factors during the laxative effects. However, the present study provides limited information since only five factors were analyzed in the serum and colon. Furthermore, multifactor analyses and mechanism studies are necessary to clarify the laxative role and mechanism of SpiA.

## 4. Materials and Methods

### 4.1. Preparation of SpiA

SpiA used in this study (Figure 1A) was kindly provided by the National Development Institute of Korean Medicine (NIKOM). It was isolated from dried roots of *Liriope platyphylla F.T. Wang & Tang (Liliaceae)* through a series of extraction steps, purification steps and nuclear magnetic resonance (NMR) analyses, as described in a previous study [6].

### 4.2. Experimental Design for Animal Study

To study the laxative effects of SpiA, the animal protocol was reviewed and approved based on the ethical procedures for scientific care guidelines by the Pusan National University-Institutional Animal Care and Use Committee (PNU-IACUC; Approval Number PNU-2017-1713). Adult ICR mice purchased from Samtako BioKorea Inc. (Osan, Korea) were handled at the Pusan National University-Laboratory Animal Resources Center, which is accredited by the Korea Food and Drug Administration (KFDA) (Accredited Unit Number-000231) and The Association for Assessment and Accreditation of Laboratory Animal Care (AAALAC) International (Accredited Unit Number; 001525). Animals were provided with *ad libitum* access to a standard irradiated chow diet (Samtako BioKorea Inc.) and water. During the experiment, all mice were maintained in a specific pathogen-free (SPF) state under a strict light cycle (lights on at 08:00 h and off at 20:00 h) at 23 ± 2 °C and 50 ± 10% relative humidity.

Constipation of ICR mice were induced based on novel methods, as described in a previous study [28]. Briefly, eight-week-old ICR mice in each group (*n* = 27) were assigned to either a nonconstipation group (untreated group, *n* = 9) or a constipation group (*n* = 18). ICR mice of the Lop-induced constipation group were further subdivided into Vehicle treated group (Lop + Vehicle treated group, *n* = 9) and SpiA treated group (Lop + SpiA treated group, *n* = 9). ICR mice were subcutaneously injected with Lop (Sigma-Aldrich, MO, USA) (4 mg/kg weight) in 0.9% sodium chloride, twice a day for 4 days. After a 3-day stationary phase, 8 mg/kg of Lop was subcutaneously administered for 4 days. Following the induction of constipation, the SpiA treated group was orally administered 20 mg/kg of SpiA, whereas the Vehicle treated group was injected with 0.9% sodium chloride alone. At 24 h after the final treatment, constipation parameters were measured in all the mice subset groups, after which all animals were euthanized using CO_2_ gas. Tissue samples were acquired and stored in Eppendorf tubes at −70 °C until further assay.

### 4.3. Analysis of Food Intake, Water Consumption and Body Weight

The food weight, water volume in bottle, and body weight of ICR mice treated with Vehicle or SpiA were measured daily at 10:00 a.m. throughout the experimental period, using an electrical balance (for food and body weight) and a measuring cylinder (for water volume). The average food intake and water consumption of each mice was then calculated using the above data. All measurements were performed in triplicate to ensure accuracy.

### 4.4. Measurement of Stool Parameters and Urine Volume

During all experimental periods, ICR mice of subset groups were bred in metabolic cages to avoid any contamination and harvest pure stools and urine (Daejong Instrument Industry Co., LTD, Seoul, Korea). The stool number and weight were measured as previously described [15,29]. Briefly, stools excreted from each ICR mice were collected daily at 10:00 am; the number of stools were counted three times, and each sample was weighed thrice using an electric balance. The water content of stool was also analyzed using the following formula:

Water content of stool = (A − B)/A × 100(1)

where, A is the weight of fresh stools collected from ICR mice of subset groups, and B is the weight of stools after drying at 60 °C for 12 h. Furthermore, urine volume was measured three times per sample using a cylinder.

### 4.5. Western Blotting Analysis

Total homogenate proteins were extracted from the colons of all subset groups (No, Lop + Vehicle and Lop + SpiA treated ICR mice) using the Pro-Prep Protein Extraction Solution (Intron Biotechnology Inc., Seongnam, Korea). Following centrifugation at 13,000 rpm for 5 min, protein concentrations were determined using a SMART^TM^ Bicinchoninic Acid Protein assay kit (Thermo Fisher Scientific Inc.). Proteins (30 μg) were then separated by 4%–20% sodium dodecyl sulfate-polyacrylamide gel electrophoresis (SDS-PAGE) for 3 h, following which the resolved proteins were transferred to nitrocellulose membranes for 2 h at 40 V. Each membrane was then incubated separately with primary antibody, overnight at 4 °C: anti-Gα (Abcam, Cambridge, UK), anti-PI-3K (Cell Signaling Technology Inc., Cambridge, MA, USA), anti-p-PI3K (Cell Signaling Technology Inc.), anti-PKC (Cell Signaling Technology Inc.), anti-p-PKC (Cell Signaling Technology Inc.), anti-MLC (Cell Signaling Technology Inc.), anti-p-MLC (Cell Signaling Technology Inc.), or anti-actin (Sigma-Aldrich Co.). The membranes were washed with washing buffer (137 mM NaCl, 2.7 mM KCl, 10 mM Na_2_HPO_4_, 2 mM KH_2_PO_4_, and 0.05% Tween 20), and incubated with horseradish peroxidase-conjugated goat anti-rabbit IgG (Zymed Laboratories, South San Francisco, CA, USA) at a dilution of 1:1000 and room temperature for 2 h. Finally, the blots were developed using a Chemiluminescence Reagent Plus kit (Pfizer Inc., Gladstone, NJ, USA). The signal band image for each protein was acquired using the digital camera (1.92 MP resolution) of the FluorChem^®^ FC2 Imaging system (Alpha Innotech Corporation, San Leandro, CA, USA). Protein densities were semiquantified using the AlphaView Program version 3.2.2 (Cell Biosciences Inc., Santa Clara, CA, USA).

### 4.6. Semi-Quantitative Reverse Transcription Polymerase Chain Reaction (RT-PCR)

Total RNA was isolated from the frozen colons using RNAzol B solution (Tel-Test Inc.), according to the manufacturer’s protocols. Following synthesis of cDNA, genes were amplified by subjecting the samples to 28 cycles of 30 s at 94 °C, 30 s at 62 °C and 45 s at 72 °C, in a Perkin-Elmer Thermal Cycler. The primer sequences used to evaluate the level of mRNA were as follows: MUC2, sense primer, 5′-GCTGC TCATT GAGAA GAACG ATGC-3′, antisense primer, 5′-CTCTC CAGGT ACACC ATGTT ACCAG G-3′; AQP8, sense primer, 5′-GTAGT ATGGA CCTAC GTGAG ATCAA GG-3′, antisense primer, 5′-AGAAC CTTTC CTCTG GACTC ACCAC C-3′; β-actin, sense and antisense primers were 5′-TGGAA TCCTG TGGCA TCCAT GAAAC-3′ and 5′-TAAAA CGCAG CTCAG TAACA GTCCG-3′, respectively. The PCR products were quantified using 1% agarose gels and a Kodak Electrophoresis Documentation and Analysis System 120.

### 4.7. Quantitative Real-Time PCR Analysis

Quantitative real-time PCR assessed the relative quantities of mRNA for MUC2 and AQ8. Total RNA molecules were isolated from frozen colon tissues using RNA Bee solution (Tet-Test Inc., Friendswood, TX, USA). After quantification of the RNA concentration, the complement DNA (cDNA) was synthesized using a mixture of oligo-dT primer (Invitrogen, Carlsbad, CA, USA), dNTP and reverse transcriptase (Superscript II, 18064-014, Invitrogen; Thermo Fisher Scientific, Inc., Waltham, MA, USA). Q-PCR was then conducted using a cDNA template and 2×Power SYBR Green (TOYOBO Co., Osaka, Japan) as described in previous studies [16]. The primer sequences used in quantitative real-time PCR was the same as the one in semi-quantitative RT-PCR analysis. The reaction cycle at which PCR products exceeded this fluorescence intensity threshold during the exponential phase of PCR amplification was considered as the threshold cycle (CT).

### 4.8. Histopathological Analysis

Colons collected from No, Lop + Vehicle and Lop + SpiA treated ICR mice were fixed with 10% formalin for 48 h, embedded in paraffin wax, and then sectioned into 4-μm thick slices which were subsequently stained with hematoxylin and eosin (H&E, Sigma-Aldrich Co.). Morphological features of the stained sections were observed by light microscopy, after which the mucosa thickness, flat luminal surface thickness and muscle thickness were measured using the Leica Application Suite (Leica Microsystems, Heerbrugg, Switzerland).

For mucin staining, colons collected from ICR mice of subset groups were fixed with 10% formalin for 48 h, embedded in paraffin wax, and then sectioned into 4-μm thick slices that were subsequently deparaffinized with xylene and rehydrated. Next, the tissue sections on the slides were rinsed with distilled water and stained with an Alcian Blue Stain kit (IHC WORLD, Woodstock, MD, USA). Finally, the morphological features in the stained colon sections were observed by light microscopy.

Immunohistochemical analysis was performed as previously described [30]. Briefly, the histological distribution of C-kit and PGP9.5 protein was observed using optical microscopy after fixing the tissue samples in 10% formalin for 48 h, embedding the tissues in paraffin, and acquiring sections 4-μm in thickness. Each section was de-paraffinized with xylene, rehydrated, and pretreated for 30 min at room temperature with a phosphate buffered saline (PBS)-based blocking buffer containing 10% goat serum. The samples were then incubated with mouse anti-C-kit (DAKO, Kyoto, Japan) and anti-PGP9.5 antibody (Abcam, Cambridge, UK), diluted 1:1000 in PBS-blocking buffer. Antigen-antibody complexes were visualized with goat anti-rabbit HRP-conjugated streptavidin secondary antibody (Histostain-Plus Kit, Zymed Laboratories) diluted 1:1000 in PBS-blocking buffer. A 3,3’-Diaminodbenzidine (DAB) substrate (Invitrogen, Carlsbad, CA, USA) and a model GS-690 imaging densitometer (Bio-Rad Laboratories, Hercules, CA, USA) were used to detect the C-kit and PGP9.5 proteins.

### 4.9. Transmission Electron Microscopy (TEM) Analysis

Colon tissues collected from 5–6 mice from each of the five treatment groups were fixed in 2.5% glutaraldehyde solution, rinsed with 1× PBS solution, dehydrated with ascending concentrations of EtOH solution, post-fixed in 1% osmium tetroxide (OsO_4_) for 1–2 h at room temperature, and embedded in Epon 812 media (Polysciences, Hirschberg an der Bergstrasse, Germany). Subsequently, ultra-thin sections of colon tissue (70-nm thick) were placed on a holey formvar-carbon coated grid, after which the grids were negatively stained using uranyl acetate and lead citrate. Morphological features of tissues were examined by TEM (Hitachi, Tokyo, Japan).

### 4.10. Measurement of Inositol Trisphosphate (IP3) Concentration

Levels of IP3 were determined using an IP3 ELISA kit (Cusabio Biotech Co., Ltd., Wuhan, China), according to the manufacturer’s instructions. Briefly, the frozen colon tissues were washed and homogenized in ice-cold PBS (pH 7.2–7.4) using a glass homogenizer (Sigma-Aldrich Co.). Tissue lysates were centrifuged at 1000 rpm for 5 min at room temperature, after which the supernatant was collected for analysis. An anti-IP3 detection antibody was added and incubated at 37 °C for 60 min, after which the substrate solution was added and the samples were further incubated for 15 min at 37 °C. The reaction was terminated following the addition of stop solution, and the plates were read at an absorbance of 450 nm using a Molecular Devices VersaMax Plate Reader (Molecular Devices, Sunnyvale, CA, USA).

### 4.11. Measurement of Gastrointestinal (GI) Hormone Concentrations

The concentration of CCK and gastrin were quantified using ELISA kits (Cusabio Biotech Co., Ltd., Wuhan, China), according to the manufacturer’s instructions. Briefly, the tissue of colons (100 mg) were homogenized in ice-cold 1× PBS (pH 7.2–7.4) using a glass homogenizer (Sigma-Aldrich Co.). The tissue lysates were then centrifuged at 1000 rpm for 5 min at 4 °C, after which the supernatant was collected for analysis. After addition of the two specific hormone antibodies (separately in each well), the supernatant was incubated for 60 min at 37 °C, to which HRP-Streptavidin solution was subsequently added and incubated for 60 min at 37 °C. This was followed by addition of the TMP One-Step Substrate Reagent, and the mixture was further incubated for 30 min at 37 °C. The reaction was terminated following addition of the stop solution. Finally, the absorbance of the reaction mixture was read at 450 nm using the Molecular Devices VersaMax Plate Reader (Sunnyvale, CA, USA).

### 4.12. AChE Activity Analysis

The AChE activity was determined using an Acetylcholinesterase Assay Kit (Abcam, Cambridge, UK), according to the manufacturer’s protocols. Briefly, the colon of each mouse was homogenized in PRO-PREP protein extraction solution (1.0 mM PMSF, 1.0 mM EDTA, 1.0 µM pepstatin, 1.0 µM leupeptin, and 1.0 µM aprotinin) (iNtRON Biotechnology Inc., Seoul, Korea), after which the homogenates were stored at −70 °C until analysis. The sample or standards and ACh reaction mixture were then incubated in a 96-well plate for 10 min at room temperature, protected from the light. Color alterations were read using a Vmax plate reader (Molecular Devices, Sunnyvale, CA, USA) at 410 nm.

### 4.13. Statistical Analysis 

Statistical significance was evaluated using one-way analysis of variance (ANOVA) (SPSS for Windows, Release 10.10, Standard Version, Chicago, IL, USA) followed by Tukey’s post hoc *t*-test for multiple comparisons. Data are presented as means ± SD (standard deviation). *p* < 0.05 is considered to indicate a statistically significant difference.

## 5. Conclusions

The results of the present study indicate that SpiA improves stool excretion, histopathological structure and cholinergic regulation in the Lop-induced constipation ICR mice model. Especially, these results provide evidence that the laxative effects of SpiA tightly correlates with the regulation of GI metabolic components and enteric nerve-related factors. These findings further indicate that SpiA could be considered as a potential prototype candidate for the treatment of constipation, although additional studies are required to confirm its mechanism of action.

## Figures and Tables

**Table 1:** Measurement of body weight, feeding behavior, stools and urine secretion in Lop-induced constipated ICR mice.

Contents		No	Lop	
			Vehicle	SpiA
Body Weight (g)		31.6 ± 2.1	29.2 ± 3.2	32.1 ± 1.8
Feeding behavior	Food intake (g/day)	2.5 ± 0.2	2.3 ± 0.4	2.4 ± 0.3
	Water consumption (mL/day)	2.4 ± 0.5	2.6 ± 0.3	2.8 ± 0.7
Stools	Number (nu)	58.5 ± 4.7	13.8 ± 3.2 *	51.7 ± 17.5 ^#^
	Weight (g)	0.54 ± 0.07	0.14 ± 0.02 *	0.61 ± 0.08 ^#^
	Water content (%)	26.9 ± 2.4	17.7 ± 1.2 *	27.5 ± 2.5 ^#^
Urine volume (mL/day)		1.4 ± 0.3	1.7 ± 0.4 *	1.3 ± 0.3 ^#^

Six-to-seven mice per group were used in body weight, feeding behavior, and secretion analyses. Each parameter was assayed in duplicate in each test. Data are reported as the mean ± SD. *, *p* < 0.05 compared to the untreated group. #, *p* < 0.05 compared to the Lop + Vehicle treated group. SpiA; Spicatoside A, Lop; loperamide, ICR; Institute of Cancer Research.

## References

[1] Sahu N.P., Banerjee S., Mondal N.B., Mandal D. (2008). Steroidal saponins. Fortschr. Chem. Org. Naturst..

[2] Escobar-Sánchez M.L., Sánchez-Sánchez L., Sandoval-Ramírez J., Ntuli T. (2015). Steroidal saponins and cell death in cancer. Cell Death—Autophagy, Apoptosis and Necrosis.

[3] Ramalingam M., Kim S.J. (2016). Insulin involved Akt/ERK and Bcl-2/Bax pathways against oxidative damages in C6 glial cells. J. Recept. Signal Transduct..

[4] Han Y., Jung H.W., Lee D.H., Kwon S.Y., Son K.H., Park Y.K. (2013). Anti-inflammatory effects of prosapogenin III from the dried roots of *Liriope platyphylla* in LPS-stimulated RAW264.7 cells. J. Asian Nat. Prod. Res..

[5] Park S.H., Lee H.J., Ryu J., Son K.H., Kwon S.Y., Lee S.K., Kim Y.S., Hong J.H., Seok J.H., Lee C.J. (2014). Effects of ophiopogonin D and spicatoside A derived from *Liriope tuber* on secretion and production of mucin from airway epithelial cells. Phytomedicine.

[6] Hur J., Lee P., Moon E., Kang I., Kim S.H., Oh M.S., Kim S.Y. (2009). Neurite outgrowth induced by spicatoside A, a steroidal saponin, via the tyrosine kinase A receptor pathway. Eur. J. Pharmacol..

[7] Rogers D.F., Barnes P.J. (2006). Treatment of airway mucus hypersecretion. Ann. Med..

[8] Voynow J.A., Rubin B.K. (2009). Mucins, mucus, and sputum. Chest.

[9] Barres B.A., Barde Y. (2000). Neuronal and glial cell biology. Curr. Opin. Neurobiol..

[10] Venkatesan R., Ji E., Kim S.Y. (2015). Phytochemicals that regulate neurodegenerative disease by targeting neurotrophins: A comprehensive review. Biol. Med. Res. Int..

[11] Wintola O.A., Afolayan A.J. (2011). Phytochemical constituents and antioxidant activities of the whole leaf extract of *Aloe ferox* Mill. Pharmacogn. Mag..

[12] Cimanga R.K., Mukenyi P.N., Kambu O.K., Tona G.L., Apers S., Totté J., Pieters L., Vlietinck A.J. (2010). The spasmolytic activity of extracts and some isolated compounds from the leaves of *Morinda morindoides* (Baker) Milne-Redh. (Rubiaceae). J. Ethnopharmacol..

[13] Najeeb-ur-Rehman, Mehmood M.H., Al-Rehaily A.J., Mothana R.A., Gilani A.H. (2012). Species and tissue-specificity of prokinetic, laxative and spasmodic effects of *Fumaria parviflora*. BMC Complement. Altern. Med..

[14] Mehmood M.H., Rehman A., Rehman N.U., Gilani A.H. (2013). Studies on prokinetic, laxative and spasmodic activities of *Phyllanthus emblica* in experimental animals. Phytother. Res..

[15] Kim J.E., Lee Y.J., Kwak M.H., Go J., Hong J.T., Hwang D.Y. (2013). Aquous extracts of *Liriope platyphylla* induced significant laxative effects on loperamide-induced constipation of SD rats. BMC Complement. Altern. Med..

[16] Choi J.Y., Kim J.E., Park J.J., Lee M.R., Song B.R., Park J.W., Kang M.J., Lee H.S., Son H.J., Hong J.T. (2018). The anti-inflammatory effects of fermented herbal roots of *Asparagus cochinchinensis* in an ovalbumin-induced asthma model. J. Clin. Med..

[17] Zhang Y.Z., Gan R.Y., Li S., Zhou Y., Li A.N., Xu D.P., Li H.B. (2015). Antioxidant phytochemicals for the prevention and treatment of chronic diseases. Molecules.

[18] Kim J.E., Yun W.B., Sung J.E., Lee H.A., Choi J.Y., Choi Y.S., Jung Y.S., Kim K.S., Hwang D.Y. (2016). Characterization the response of Korl:ICR mice to loperamide induced constipation. Lab. Anim. Res..

[19] Kim J.E., Go J., Koh E.K., Song S.H., Sung J.E., Lee H.A., Lee Y.H., Hong J.T., Hwang D.Y. (2016). Gallotannin-enriched extract isolated from Galla Rhois may be a functional candidate with laxative effects for treatment of loperamide-induced constipation of SD rats. PLoS ONE.

[20] Lee Y.K., Kim J.E., Nam S.H., Goo J.S., Choi S.I., Choi Y.H., Bae C.J., Woo J.M., Cho J.S., Hwang D.Y. (2011). Differential regulation of the biosynthesis of glucose transporters by the PI3-K and MAPK pathways of insulin signaling by treatment with novel compounds from Liriope platyphylla. Int. J. Mol. Med..

[21] Wintola O.A., Sunmonu T.O., Afolayan A.J. (2010). The effect of *Aloe ferox* Mill. in the treatment of loperamide-induced constipation in Wistar rats. BMC Gastroenterol..

[22] Kim J.E., Go J., Lee H.S., Hong J.T., Hwang D.Y. (2018). Spicatoside A in red *Liriope platyphylla* displays a laxative effect in a constipation rat model via regulating mAChRs and ER stress signaling. Int. J. Mol. Med..

[23] Muller P.Y., Milton M.N. (2012). The determination and interpretation of the therapeutic index in drug development. Nat. Rev. Drug Discov..

[24] Kim J.E., Lee M.R., Park J.J., Choi J.Y., Song B.R., Son H.J., Choi Y.W., Kim K.M., Hong J.T., Hwang D.Y. (2018). Quercetin promotes gastrointestinal motility and mucin secretion in loperamide-induced constipation of SD rats through regulation of the mAChRs downstream signal. Pharm. Biol..

[25] Yang Z.H., Yu H.J., Pan A., Du J.Y., Ruan Y.C., Ko W.H., Chan H.C., Zhou W.L. (2008). Cellular mechanisms underlying the laxative effect of flavonol naringenin on rat constipation model. PLoS ONE.

[26] Wang Y., Liu Y., Wang G., Han L., Xia T., Liu Z., Man S., Gao W., Liue C. (2017). Effects of *Rhizoma parisdis* total saponins and its main compounds on gastric emptying via regulating muscarinic receptors in vitro and in vivo. RSC Adv..

[27] Nezami B.G., Srinivasan S. (2010). Enteric nervous system in the small intestine: Pathophysiology and clinical implications. Curr. Gastroenterol. Rep..

[28] Suo H., Zhao X., Qian Y., Li G., Liu Z., Xie J., Li J. (2014). Therapeutic effect of activated corbon-induced constipation mice with *Lactobacillus fermentum* Suo on treatment. Int. J. Mol. Sci..

[29] Yan S., Yue Y., Wang X., Dong H., Zhen S., Wu B., Qian H. (2017). Aqueous extracts of *Herba Cistanche* promoted intestinal motility in loperamide-induced constipation rats by ameliorating the interstitial cells of cajal. Evid. Based Complement. Altern. Med..

[30] Yin J., Liang Y., Wang D., Yan Z., Yin H., Wu D., Su Q. (2018). Naringenin induces laxative effects by upregulating the expression levels of c-Kit and SCF, as well as those of aquaporin 3 in mice with loperamide-induced constipation. Int. J. Mol. Med..

